# Emodin induces apoptosis of human cervical cancer hela cells via intrinsic mitochondrial and extrinsic death receptor pathway

**DOI:** 10.1186/1475-2867-13-71

**Published:** 2013-07-16

**Authors:** Wang Yaoxian, Yu Hui, Zhang Yunyan, Liu Yanqin, Ge Xin, Wu Xiaoke

**Affiliations:** 1 Department of Gynecology, Third Affiliated Hospital of Harbin Medical University, Hapin Road, 150086, Haerbin, Heilongjiang Province, China; 2 Department of Gynecology, First Affiliated Hospital of Heilongjiang University of Chinese Medcine, Hapin Road, 150040, Haerbin, Heilongjiang Province, China; 3 Cardiopulmonary Function Room, Third Affiliated Hospital of Harbin Medical University, Hapin Road, 150086, Haerbin, Heilongjiang Province, China; 4 Department of General Surgery, Heilongjiang Province’s Hospital, Hapin Road, 150036, Haerbin, Heilongjiang Province, China

**Keywords:** Emodin, HeLa cells, Tunel, Flow cytometry, Apoptosis

## Abstract

**Background:**

Emodin is a natural anthraquinone derivative isolated from the Rheum palmatum L. Aim: The aim of the present study was to investigate the effect of emodin on the apoptosis of the human cervical cancer line HeLa and to identify the mechanisms involved.

**Methods:**

Relative cell viability was assessed by MTT assay after treatment with emodin. Cell apoptosis was detected with TUNEL, Hoechst 33342 staining and quantified with flow cytometry using annexin FITC-PI staining.

**Results:**

The percentage of apoptotic cells was 0.8, 8.2, 22.1, and 43.7%, respectively. The mRNA levels of Caspase-9, -8 and −3 detected by Real-time PCR after treatment with emodin were significantly increased. Emodin increased the protein levels of Cytochome *c*, Apaf-1, Fas, FasL, and FADD but decreased the protein levels of Pro-caspase-9, Pro-caspase-8 and Pro-caspase-3.

**Conclusion:**

We conclude that the emodin inhibited HeLa proliferation by inducing apoptosis through the intrinsic mitochondrial and extrinsic death receptor pathways.

## Introduction

Cervical cancer is the second female cancer worldwide as the most common malignancy in both incidence and mortality [1]. More than 80% of cases are found in developing countries [2]. There are several treatments used for cervical cancer, but each of them has apparent drawbacks. Surgical treatment is restricted only for patients with early stage and the young patients who have lost fertility [3]. Radiotherapy and chemotherapy are not specific to cancer cells and often bring severe adverse effect, including bone marrow suppression, nerve injury, gastrointestinal adverse reactions, renal impairment and second cancer occurrence [3]. Although the technology and method become more and more advanced, up to 35% patients will still develop persistent/recurrence/metastatic disease when the treatment results are poor. New therapeutic strategies must be evaluated to improve survival. Thus, finding a safer and more efficient treatment remains an arduous task.

Recent studies have focused on the anti-tumor properties of natural products because these medicines have fewer side-effects and are more suitable for long-term use compared with chemically synthesized medicines. Emodin (1, 3, 8-trihydroxy-6-methyl-anthraquinone), a naturally occurring anthraquinone, present in the roots and barks of numerous plants, is an active ingredient of various Chinese herbs including *Rheum officinale* and *Polygonam cuspidatum*medicine [4]. Pharmacological studies have demonstrated that emodin possesses anti-bacterial [5], anti-inflammatory [6], immuno-suppressive [7], and anti-cancer effects [8]. In vitro and in vivo studies have demonstrated its potential as an excellent cytotoxicity against a variety of malignant human cancers such as lung cancer [9], chronic myelocytic leukemia [10], liver cancer [11], tongue squamous cancer [12], gastric cancer [13], prostate cancer [14] and gallbladder cancer [15] but emodin have almost no toxic effect on normal cells [16,17]. However, the molecular mechanisms for the growth inhibition and cytotoxicity of emodin-treated HeLa cells are poorly understood.

In recent years, apoptosis has emerged as the major mechanism by which anticancer agents eliminate preneoplastic or neoplastic cells. It has been proven that emodin can induce apoptosis through increasing nuclear condensation and DNA fragmentation [18,19], activating caspase −9 and −3 [20], inducing cell-cycle arrest [12,21], elevating level of ROS [22,23], decreasing level of NF-κB [24,25], activating PI3K/AKT pathway [26] and PKC pathway [20,27], However, there is no available information to address how emodin affects human cervical cancer cells in vitro. The aim of the present study is to investigate the potential anticancer effects of emodin on human cervical cancer cells and the underlying molecular mechanisms.

## Materials and methods

### Chemicals and reagents

Emodin and MTT [3-(4, 5-dimethylthiazol-2-yl)-2, 5-diphenyltetrazolium bromide] were obtained from Sigma Chemical Co. (USA). The primers of caspase-8, -9 and −3 for Real-time PCR were purchased from Genscript (China). Antibodies against Cytochrome *c*, Apaf-1, Caspase-9, Fas, FasL, FADD, Caspase-8, Caspase-3, Gapdh and β-actin were purchased from Cell Signaling Technology (USA). Fluorescence-conjugated secondary antibodies were purchased from Invitrogen (USA). Other chemicals were obtained in their commercially available highest purity grade.

### Cell culture

The human cervical cancer HeLa cells were obtained from American Type Culture Collection (ATCC). Cells were cultured in Dulbecco’s modified Eagle’s medium supplemented with 10% (v/v) fetal calf serum, 100 μg/mL streptomycin, and 100U/mL penicillin. Cultures were maintained at 37°C in a humidified incubator in an atmosphere of 5% CO_2_.

### MTT assay for cell proliferation

Cell proliferation was determined by MTT assay. In brief, the HeLa cells in logarithmic phase were seeded into 96-well plate at 1×10^4^cells/well followed by incubation at 37°C for 24 h for attachment and then treated with emodin (0, 10, 20, 30, 40 and 50 μM) for 24, 48 or 72 h, respectively. Six wells were included in each group. 20 μL of 5 mg/mL MTT dye was added to each well and incubated at 37°C for 4 h. Then the supernatant was discarded and purple-colored precipitates of formazan were dissolved by gently shaking for 10 min in 150 μL of dimethyl sulfoxide (DMSO). After complete dissolution, absorbance (A) was measured at 490 nm on a microplate reader. The effect of emodin on growth inhibition was assessed as the percentage of inhibition in cell growth. Background absorbance of the medium in the absence of cells was subtracted. Percent viability was calculated as [value of drug-treated group (A)/control group (A)] × 100%. Each assay was carried out three times, and the results were expressed as the mean (± SEM). Similar results were observed in at least three independent experiments.

### Detection of apoptotic cells by TUNEL and Hoechst 33342 staining

The apoptotic HeLa cells were detected using the TUNEL assay that was performed using an *in Situ* Nick-End Labeling kit (Beyotime Institute of Biotechnology, China). Cells were treated with emodin (0, 20, 40 and 80 μM) in 96-well plates. After 48 h, the attached cells were washed with PBS and then fixed in freshly prepared 4% paraformaldehyde for 30 min, then washed with PBS and incubated with digoxigenin-conjugated dUTP in a terminal deoxynucleotidyl transferase-catalyzed reaction for 1 h at 37°C in a humidified atmosphere. After the cells were immersed in stop/wash buffer for 10 min at room temperature and washed with PBS, they were incubated with an anti-digoxigenin antibody conjugating peroxidase for 30 min. The nuclei fragments were stained using 3, 3’-diaminobenzidine (DAB) as a substrate of the peroxidase for 5 min. Apoptotic cells were stained brown.

The apoptosis of HeLa cells was also detected using the Hoechst 33342 assay kit (Beyotime Institute of Biotechnology, China). The HeLa cells were seeded on coverslips on a 6-well plate and treated with emodin (0, 20, 40 and 80 μM). After 48 h, the attached cells were washed with PBS and fixed in freshly prepared 4% paraformaldehyde for 30 min, then washed with PBS and incubated with Hoechst 33342 staining solution for 5 min. After treatment, cells were washed with PBS and added Antifade Mounting Medium, then detected the apoptosis by fluorescence microscope. Apoptosis, with condensed and fragmented nuclei, was observed under fluorescence microscope.

### Quantification of apoptosis by flow cytometry

The apoptotosis of HeLa cells was quantified using flow cytometry. After incubation with emodin (0, 20, 40 and 80 μM) in six-well plates for 48 h, the cells were harvested with trypsin treatment and centrifugation, washed with PBS, stained with 10 μL annexin V-FITC and 5 μL propidium iodide (PI) in the dark at room temperature for 15 min according to the manufacturer’s protocol (Biosea, China) and then analyzed with Becton FACSC flow cytometer (Becton Dickinson Corporation, USA). For each condition, 1×10^4^ cells were studied in each cytometry experiment.

### RNA isolation and real-time PCR analysis

Total RNA was extracted by a Trizol reagent kit (Invitrogen, USA) after the HeLa cells treated with emodin (0, 20, 40 and 80 μM) for 48 h. The quality of each RNA sample (including its concentration and purity) was checked by measuring the absorbance. One microgram RNA from each sample was used to generate cDNA using M-MLV reverse transcriptase as per manufacturer's specifications (Promega Corporation, USA). After an initial denaturation step at 95°C for 10 min using SYBR Green PCR Master Mix (Applied Biosystems, USA), Real-time PCR was cycled 40 times between 95°C /15 s and 60°C /1 min. Amplification was performed using 7500 Fast Real-Time PCR Systems (Applied Biosystems, USA) and the products were routinely checked using dissociation curve software. Transcript quantities were compared by the relative Ct method and the amount of Caspase-9, -8 and −3 were normalized to the endogenous control (GAPDH). The value in relation to the control sample was given by 2-∆∆CT. Real-time PCR primer sequences for caspases measurements were as following:

Caspase 9: sense: 5′-CGAACTAACAGGCAAGCAGC-3′ anti-sense: 5′-ACCTCACCAAATCCTCCAGAAC-3′;

Caspase 8: sense: 5′-GCCTCCCTCAAGTTCCT-3′ anti-sense: 5′-CCTGGAGTCTCTGGAATAACA-3′;

Caspase 3: sense: 5′-TGGTTCATCCAGTCGCTTTG-3′ anti-sense: 5′-CATTCTGTTGCCACCTTTCG-3′.

### Western blot analysis

Following treated with emodin (0, 20, 40 and 80 μM) for 48 h, HeLa cells were washed with ice-cold PBS and collected in lysis buffer including 50 mM Tris, pH 7.4, 150 mM NaCl, 1% NP-40, 0.25% sodium deoxycholate, 0.1% SDS, 1 mM Na_3_VO_4_, 1 mM NaF, 1 mM EDTA, 1 mM PMSF and 1μg/mL leupeptin. The supernatant was obtained by centrifuging at 13,500 rpm for 20 min. Total protein was extracted and protein concentration was determined by Bradford assay. For immunoblotting, 120 μg proteins from each sample were subjected to electrophoresis on 12% SDS-PAGE and separated proteins were transferred onto a PVDF membrane. The PVDF membrane was blocked with 5% non-fat milk powder (w/v) at room temperature for 2 h, then incubated with the primary antibodies against Cytochrome c (1:500), Apaf-1 (1:500), Caspase-9 (1:500), Fas (1:500), FasL (1:500), FADD (1:500), Caspase-8 (1:500), Caspase-3 (1:500), Gapdh (1:1000), and β-actin (1:500), respectively, at 4°C overnight. After washing, the membrane was incubated with fluorescence-conjugated secondary antibody (anti-rabbit or anti-mouse, 1:10000; Invitrogen, USA) at room temperature for 50 min. Gapdh or β-actin was used as an internal control to monitor equal protein loading and transfer of proteins from the gel to the membranes after stripping them with the Gapdh and β-actin antibodies. Western blot bands were quantified using the Odyssey infrared imaging system (LI-COR, USA). All results represent of three independent experiments.

### Statistical analysis

Data were reported as means ± SEM of at least three independent experiments. For statistical analysis, one-way ANOVA was used for comparison of one variance among groups and two-way ANOVA was used for comparison of two independent variances among groups followed by the Tukey *post hoc* test. A P value less than 0.05 was considered to be significant.

## Results

### Emodin-induced morphological changes and anti-proliferation of HeLa cells

**Figure 1:**
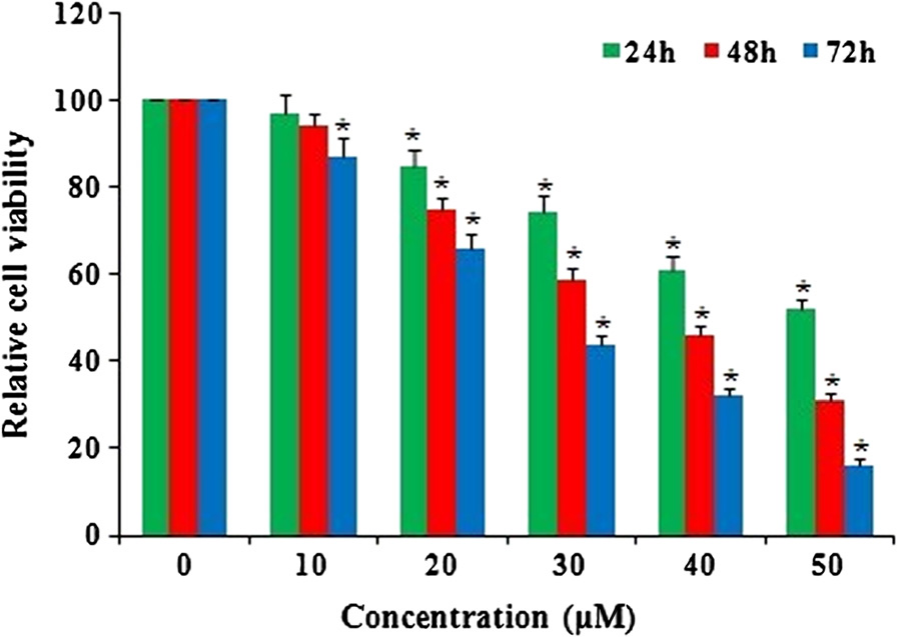
**Emodin-induced anti-proliferation of HeLa cells.** HeLa cells were treated with emodin at doses of 0, 10, 20, 30, 40, and 50 μM for 24, 48, and 72 h. Cell viability was evaluated with the MTT assay and results are reported as relative cell viability (%). All data were normalized to the control group which was considered to be 100%. The result showed that emodin inhibited proliferation of HeLa cells in a dose- and time-dependent manner. *P<0.05 versus control group (0 μM) (two-way ANOVA followed by the Tukey *post hoc* test).

The morphology of the HeLa cells was examined using a phase contrast microscope. In the presence of emodin, HeLa cells showed round morphology with small shrinkage and nuclear condensation, a proportion of the cells revealed swelling, cell membrane lysis and disintegration of organelles, suggesting emodin-induced toxicity to HeLa cells (pictures not shown). HeLa cells were incubated with emodin (0, 10, 20, 30, 40 and 50 μM) and cell viability was evaluated by the MTT assay at 24, 48 and 72 h. Treatment with 10, 20, 30, 40 and 50 μM emodin significantly reduced cell viability compared to the control group (Figure 1), indicating a dose-dependent effect of emodin on cell viability. Among all the tests, cells incubated with 50 μM emodin for 72 h showed the maximum anti-proliferation effect, with cell viability decreased to 16% of the control cells. These results suggest that emodin inhibits proliferation of HeLa cells in a dose- and time-dependent manner.

### Emodin-induced apoptosis of HeLa cells

**Figure 2:**
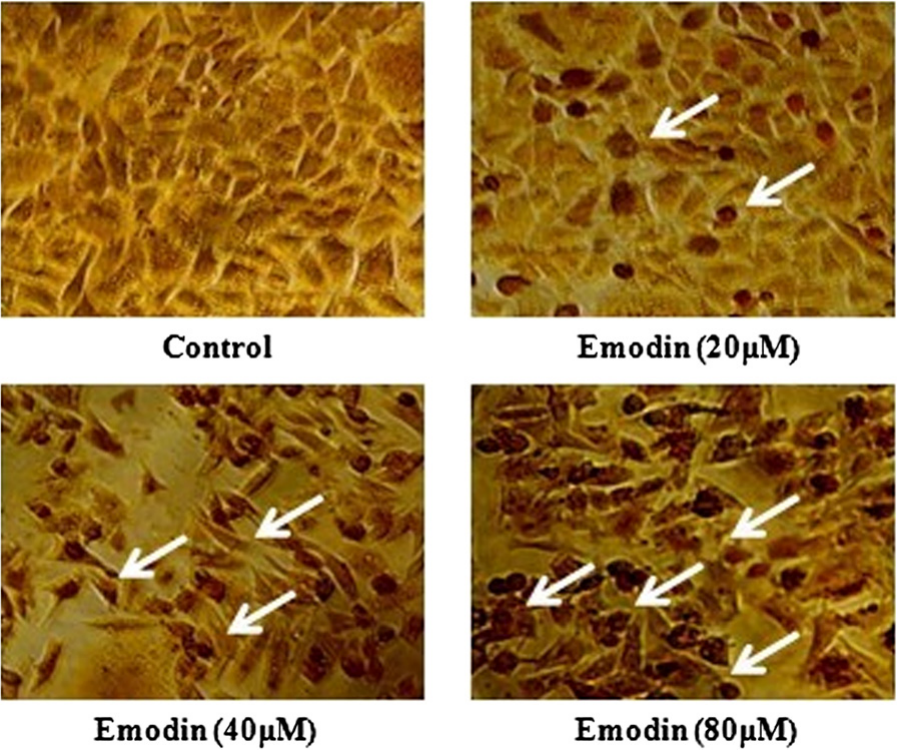
**Cell apoptosis observed using TUNEL staining.** HeLa cells were treated with Emodin (0, 20, 40, and 80 μM) for 48 h. Apoptotic cells exhibited Morphological changes in the nuclei typical of apoptosis. Photographs were taken under an inverted microscope (200**×**, original magnification). Arrows represent apoptotic cells.

**Figure 3:**
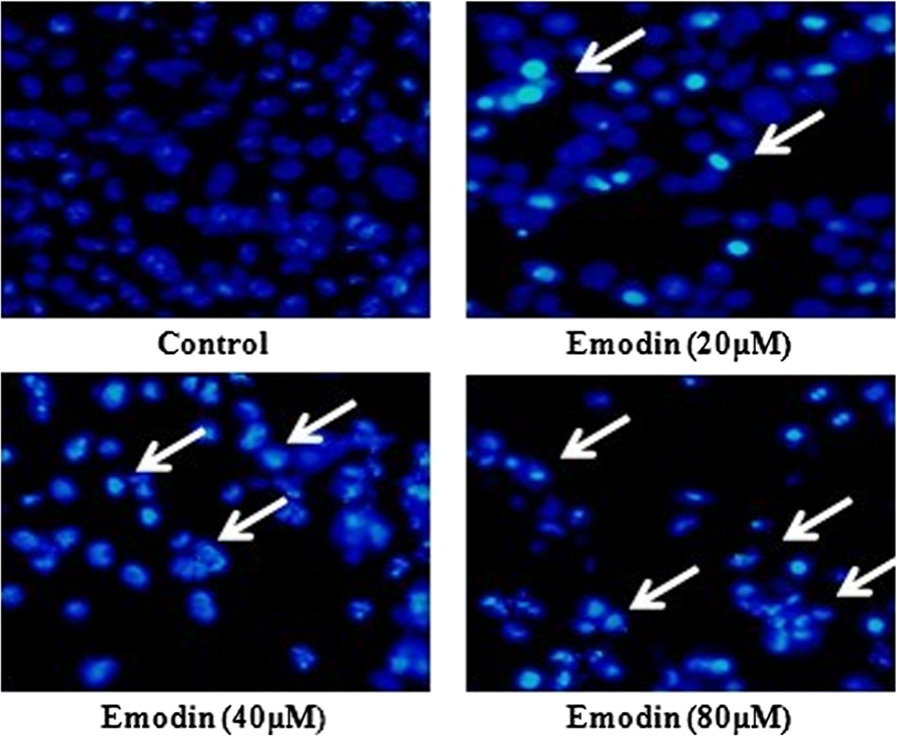
**Cell apoptosis observed using Hoechst 33342 staining.** HeLa cells were treated with Emodin (0, 20, 40, and 80 μM) for 48 h. Apoptotic cells exhibited Morphological changes in the nuclei typical of apoptosis. Photographs were taken under a fluorescence microscope (200**×**, original magnification). Arrows represent apoptotic cells.

The TUNEL and Hoechst 33342 apoptosis detection kit were used after cells were treated with emodin (0, 20, 40 and 80 μM) for 48 h. Representative images of TUNEL and Hoechst 33342 staining were shown in Figures 2 and 3. The number of apoptotic HeLa cells (white arrows) increased with the dose of emodin. Apoptotic HeLa cells displayed a round and shrunken cell body, suggesting that emodin-induced apoptosis of HeLa cells might contribute to reduced cell viability.

**Figure 4:**
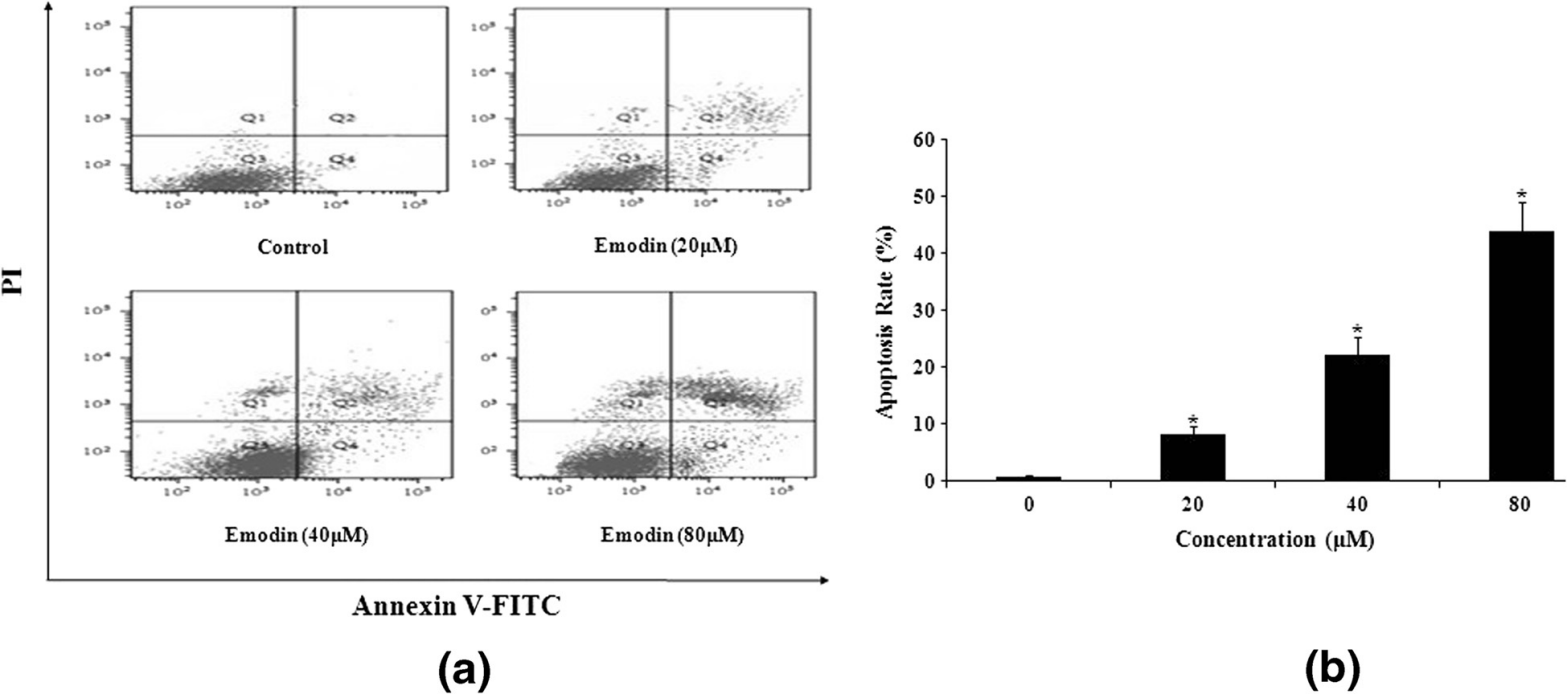
**Emodin-induced apoptosis in HeLa cells was determined by flow cytometry using annexin FITC-PI staining method.** The cells were treated with Emodin (0, 20, 40, and 80 μM) for 48 h **(a, b)**. **(a)** Emodin-induced apoptosis analyzed by flow cytometry. The lower right quadrant (Q4) indicates the percentage of early apoptotic cells (FITC-stained cells) and the upper right quadrant (Q2) indicates the percentage of late apoptotic cells (FITC+PI-stained cells). **(b)** Emodin-induced apoptosis rate shown by bar graph. The experiment was repeated three times and the percentage of apoptotic cells (means± SEM) for each treatment group is shown in b. *P<0.05 versus control group (0 μM) (one-way ANOVA followed by the Tukey *post hoc* test).

To further quantify emodin-induced apoptosis of HeLa cells, cells were stained with annexin V-FITC and PI, followed by flow cytometry. A representative result of flow cytometry was presented in Figure 4a. The lower right quadrant (Q4) depicts the percentage of early apoptotic cells (annexin V-FITC-stained cells) and the upper right quadrant (Q2) represents the percentage of late apoptotic cells (annexin V-FITC and PI-stained cells). The fully apoptotic cells are those in the lower right and upper right quadrants. As shown in the quantitative result in Figure 4b, only a small number of apoptotic cells was detected in the control group. However, 48 h after treatment with 0, 20, 40 and 80 μM emodin, cell apoptosis was 0.8, 8.2, 22.1, and 43.7%, respectively. These results suggest that emodin induced significant apoptosis of HeLa cells in a dose-dependent manner.

### Emodin increased mRNA expression of caspase-8, -9 and 3

**Figure 5:**
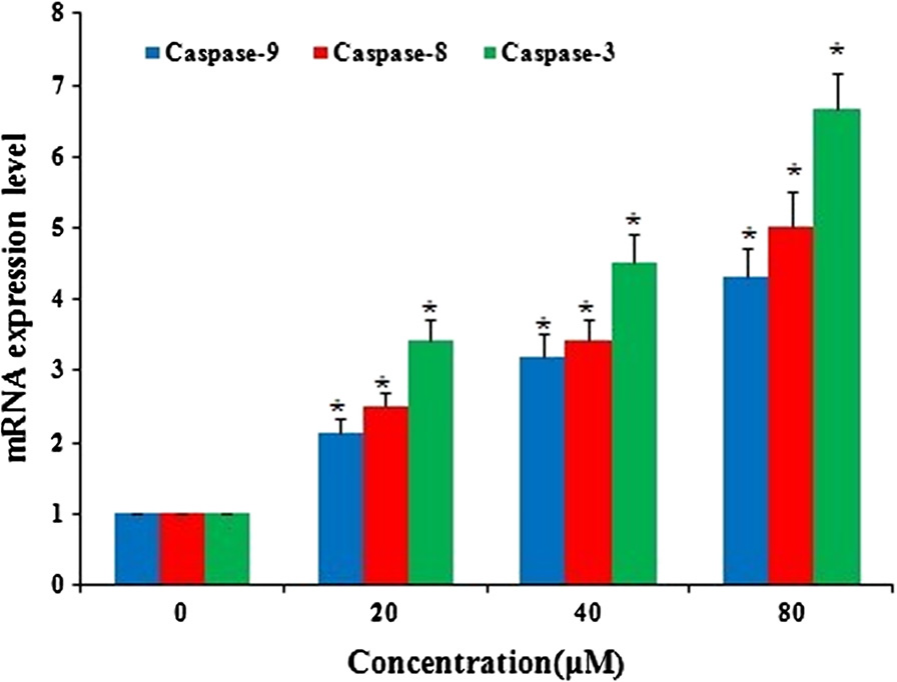
**Emodin increased gene expression of caspase-9, -8, and −3 in HeLa cells in a dose-dependent manner.** HeLa cells were treated with Emodin (0, 20, 40 and 80 μM) for 48 h. The expression of mRNAs was analyzed by Real-time quantitative PCR and normalized by GAPDH expression. *P<0.05 versus control group (0 μM) (two-way ANOVA followed by the Tukey *post hoc* test).

Real-time quantitative PCR was used to detect the mRNA expression of Caspase-9, -8 and −3 at 48 h after emodin treatment with emodin (0, 20, 40 and 80 μM). The change of mRNA expression was normalized by GAPDH expression. The result showed that the mRNA expression of Caspase-9, -8 and −3 increased significantly after treatment with emodin for 48 h and the up-regulation exhibited an emodin dose-dependent pattern (Figure 5).

Emodin increased Cytochome *c*, Apaf-1 but decreased Pro-caspase-9 in intrinsic mitochondrial pathway and increased Fas, FasL, FADD but decreased Pro-caspase-8 in extrinsic death receptor pathway in HeLa cells.

To further elucidate the molecular mechanism underlying the emodin-induced apoptosis in HeLa cells, we examined the related protein expressions of the intrinsic mitochondrial pathway and the extrinsic death receptor pathway about apoptosis by Western blot.

**Figure 6:**
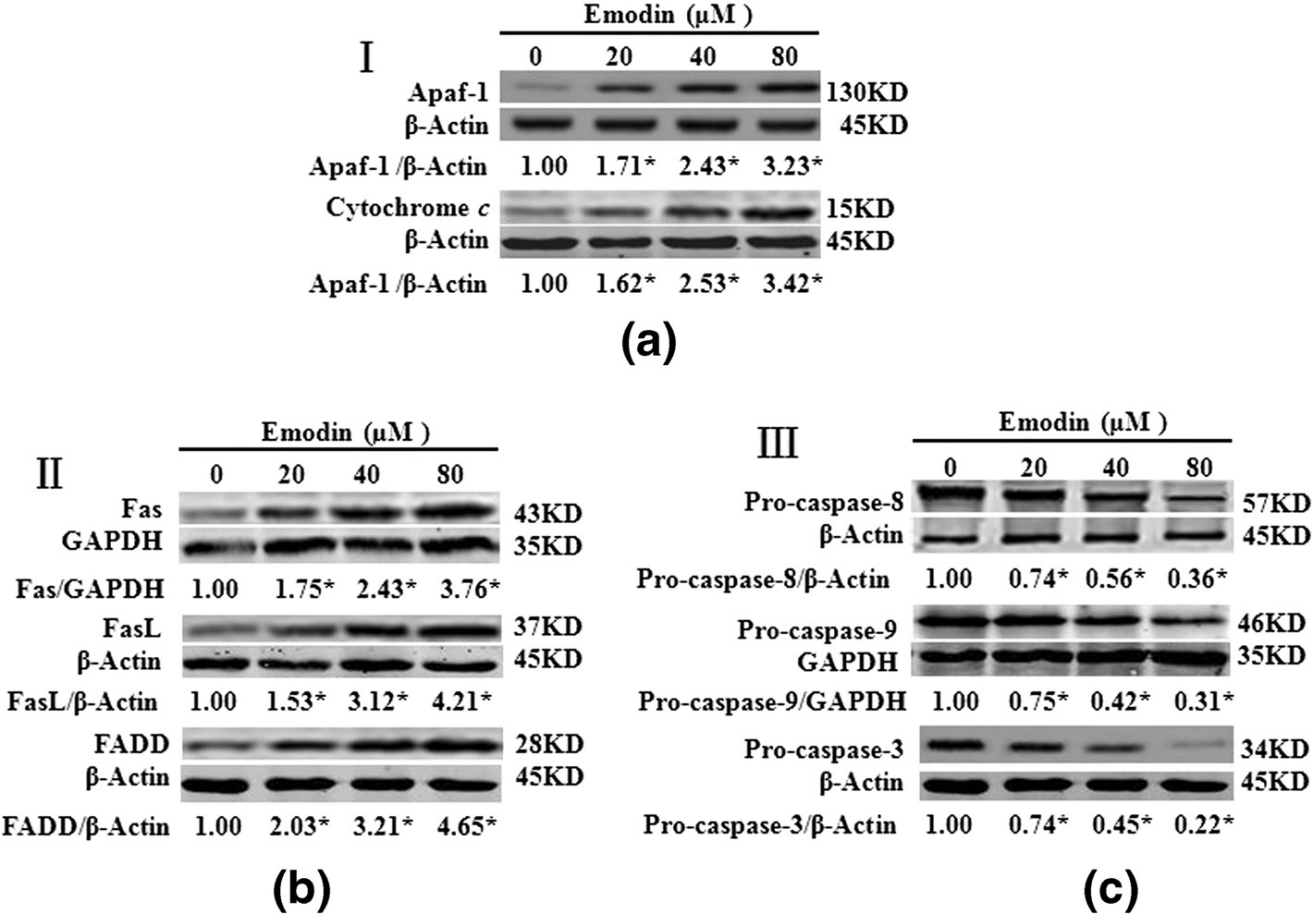
**Emodin increased the expression of Apaf-1, Cytochrome c, Fas, FasL, and FADD but decreased the expression of Pro-caspase-9, Pro-caspase-8, and Pro-caspase-3 in HeLa cells.** HeLa cells were treated with Emodin (0, 20, 40 and 80 μM) for 48 h and the expression of proteins in treated cells was determined by Western blot analysis. (**a**), Emodin increased the expression of Apaf-1, Cytochrome *c*. (**b**), Emodin increased the expression of Fas, FasL, and FADD. (**c**), Emodin decreased the expression of Pro-caspase-8, Pro-caspase-9, and Pro-caspase-3. Data are reported as the means±SEM of at least three experiments. *P<0.05 versus control group (0 μM) (two-way ANOVA followed by the Tukey *post hoc* test).

Western blot analysis was used to further detected protein expressions of Cytochomec, Apaf-1, Caspase-9, Fas, FasL, FADD, Caspase-8, and Caspase-3 in HeLa cells after emodin (0, 20, 40 and 80 μM) treatment for 48 h. The GAPDH or β-actin was used as an internal loading control. On one hand, in the present study, emodin treatment increased Cytochromec and Apaf-1 protein expression while it decreased Pro-caspase-9 and Pro-caspase-3 protein expression in treated HeLa cells. The quantitative results showed emodin increased the protein levels of Cytochrome *c* and Apaf-1 but decreased the protein levels of Pro-caspase-9 and Pro-caspase-3 in a dose-dependent manner and the results were 3.23, 3.42 and 0.36, 0.22 folds of the control level at 80 μM (Figure 6a and 6c). On another hand, emodin treatment increased Fas, FasL, FADD protein expression while it decreased Pro-caspase-8 and Pro-caspase-3 protein expression in treated HeLa cells. The quantitative results showed emodin increased the protein levels of Fas, FasL, FADD but decreased the protein levels of Pro-caspase-8 and Pro-caspase-3 in a dose-dependent manner and the results were 3.76, 4.21, 4.65 and 0.31, 0.22 folds of the control level at 80 μM (Figure 6b and 6c). The effect of emodin on regulating the expression of apoptosis-related proteins further supported the observation of emodin-induced apoptosis in HeLa cells.

## Discussion

In this study, we have examined the effect of emodin on human cervical cancer cell line HeLa. We observed a dose- and time-dependent anti-proliferation effect of emodin on these cells. The emodin-induced apoptosis might be mediated by the activation of intrinsic mitochondrial pathway and extrinsic death receptor pathway through regulation the expression of related apoptotic factors.

The effect of emodin-induced apoptosis has been previously reported in other cell types [18-27]. It is well known that cell death can be divided into necrosis and apoptosis [28]. Apoptosis is a highly regulated, organized and programmed cell death process controlling the development and homeostasis of multicellular organisms [29], it could kill cancer cells without causing damage to normal cells or surrounding tissues [30]. Thus, induction of apoptosis in cancer cells is a key mechanism by which anticancer therapy works [31]. In this study, we also observed an anti-proliferation effect of emodin on HeLa cells by the induction of apoptosis and this effect exhibited a dose- and time-dependent pattern.

There are two major pathways that could induce apoptosis: the intrinsic mitochondria pathway and extrinsic death receptor pathway [32,33]. In the intrinsic pathway, many factors such as environmental changes, stimuli and drugs could induce mitochondria dysfunction. Cytochrome c is released from dysfunctional mitochondria and accumulated in the cytoplasm where it binds to the protein Apaf-1, meanwhile, binding of Pro-caspase-9 to Apaf-1 oligomers results in the formation of apoptosome, eventually leading to the activation of caspase-3, DNA damage and cell apoptosis [34-39]. Many previous studies have shown that emodin inhibits proliferation and induces apoptosis in many carcinoma cells via intrinsic mitochondria pathway [18-20,22,23,40,41]. Our data also showed that emodin induced up-regulation of Cytochome *c* and Apaf-1 but down-regulation Pro-caspase-9 and Pro-caspase-3 in HeLa cells, suggesting the involvement of the intrinsic mitochondria pathway in emodin-induced apoptosis also happened in HeLa cells.

The extrinsic death receptor pathway involves Fas, TNFR1, DR3, DR4 and DR5. In these factors, Fas and FasL have been regarded as very important effectors of apoptosis in various biological conditions and its disregulated expression in a variety of carcinomas such as breast [42], hepatocellular [43], colorectal [44], and nasopharyngeal [45] carcinoma. Fas (CD95 or APO-1) [46], is a 36-kDa cell surface protein that belongs to the death receptor (DR) family. Activation of Fas with its natural ligand FasL, induces apoptosis in sensitive cells [46]. The Fas-mediated cell death pathway includes cell death transactivation adaptor molecular (FADD) with a death domain and a FADD-associated Pro-caspase-8 that forms death inducing signaling complex (DISC) resulting in apoptotic cell death [47]. Meanwhile, Pro-caspase-8 binds to Fas-bound FADD leading to the activation of Caspase-8 [46], and then leads to the activation of Caspase-3 [48]. This caspase cascade leads DNA degradation, and ultimately cell death [49-52]. Our data showed that emodin induced up-regulation of Fas, FasL, FADD but down-regulation Pro-caspase-8 and Pro-caspase-3 in HeLa cells, these results further support the apoptotic effect of emodin on HeLa cells also via extrinsic death receptor pathway.

In this study, we could conclude that intrinsic mitochondrial pathway and extrinsic death receptor pathway were involved in anti-tumor effect of emodin in HeLa cells. Emodin has been long used by Chinese people as an oral medicine and has been proven to be an effective medicine possesses anti-bacterial, anti-inflammatory, immuno-suppressive, and anti-cancer effects. The anti-tumor effect of emodin on cervical cancer in vivo and the possible underlying molecular mechanism require further study, while emodin has the potential to be developed as a chemotherapeutic or adjuvant agent for human cervical cancers.

## Conclusion

In short, we concluded that the emodin inhibited HeLa proliferation by inducing apoptosis through the intrinsic mitochondrial and extrinsic death receptor pathways.

## Competing interest

The authors declare that there are no conflicts of interest.

## Authors’ contributions

WY carried out the studies, YH, ZY, LY, GX participated in the studies. WY and WX conceived of the study, and participated in its design and coordination and helped to draft the manuscript. All authors read and approved the final manuscript.
